# Supplementary figures and images for: The complete mitochondrial genome of Cycas debaoensis revealed unexpected static evolution in gymnosperm species
Source: PLoS One. 2021 Jul 22;16(7):e0255091. doi: 10.1371/journal.pone.0255091 (PMC8297867; doi:10.1371/journal.pone.0255091)

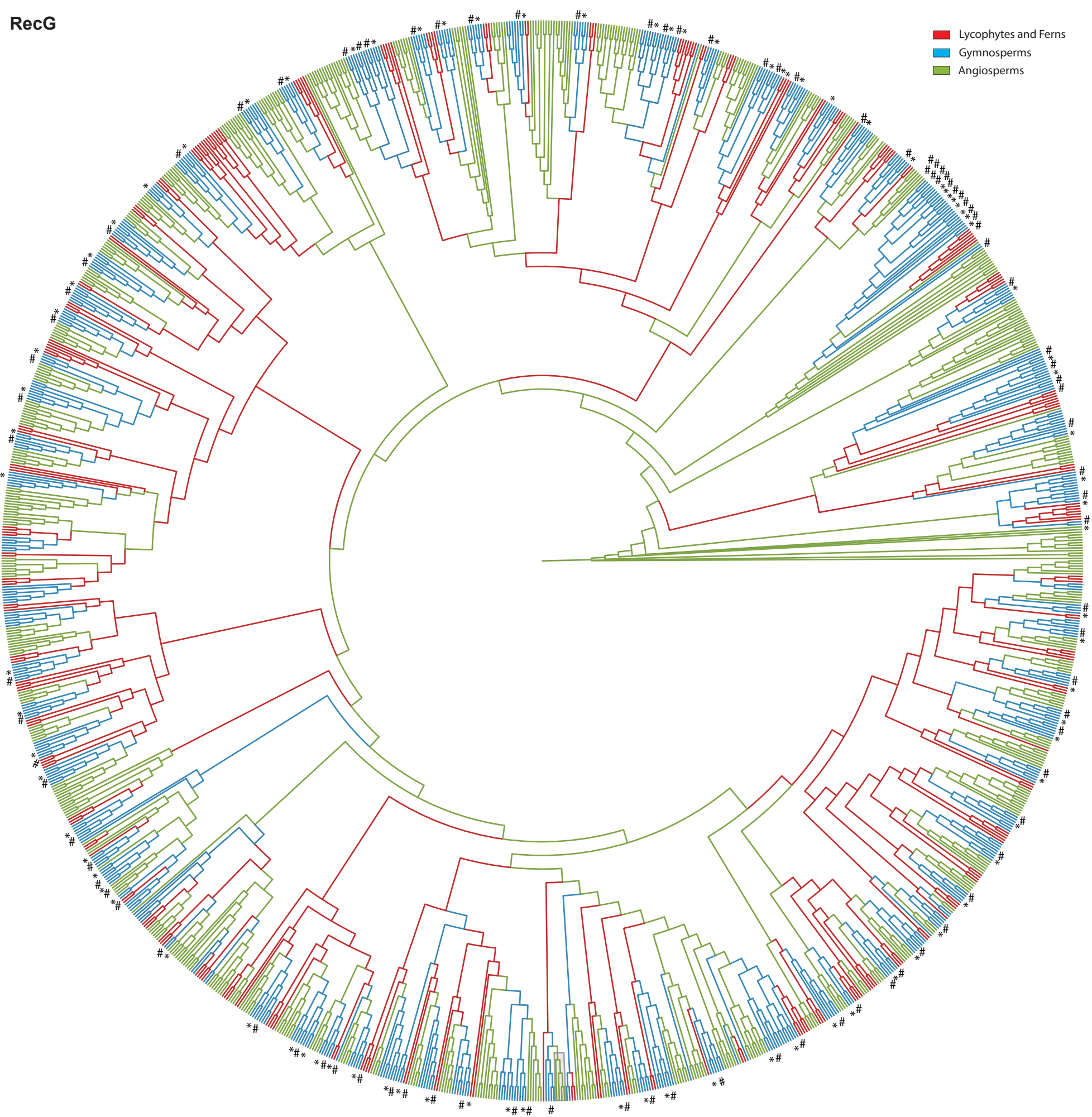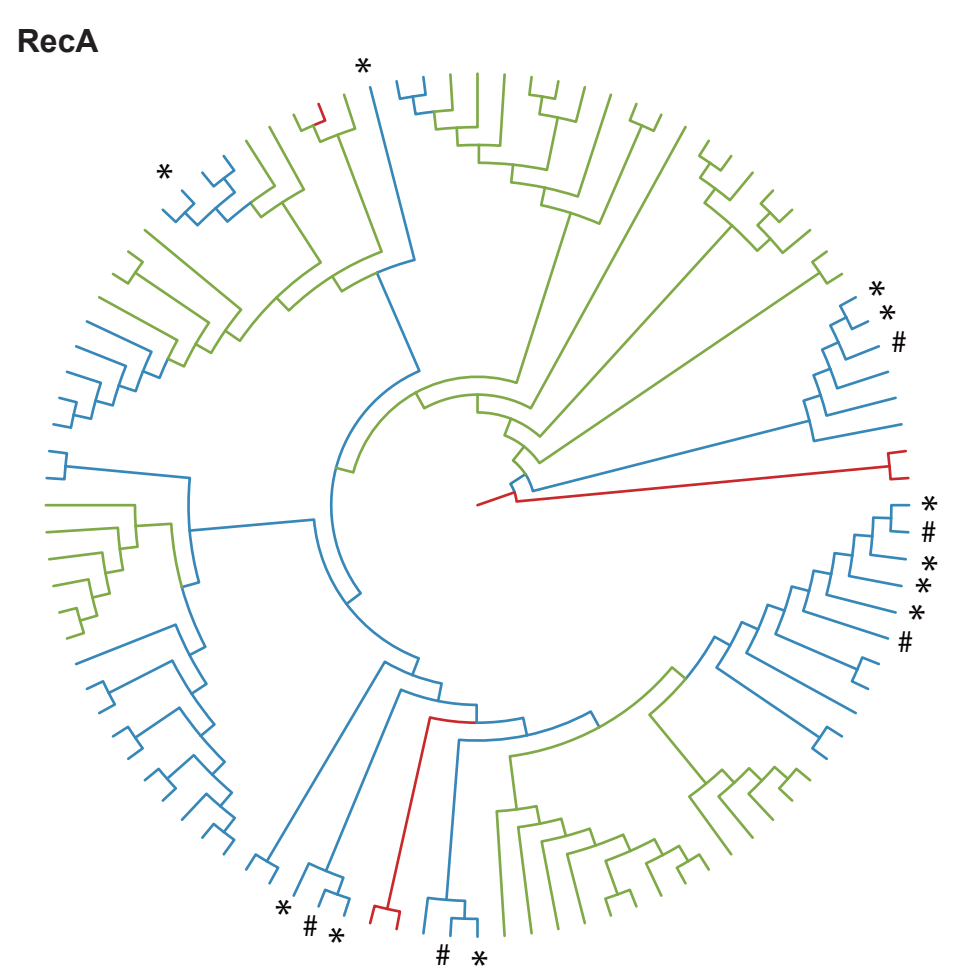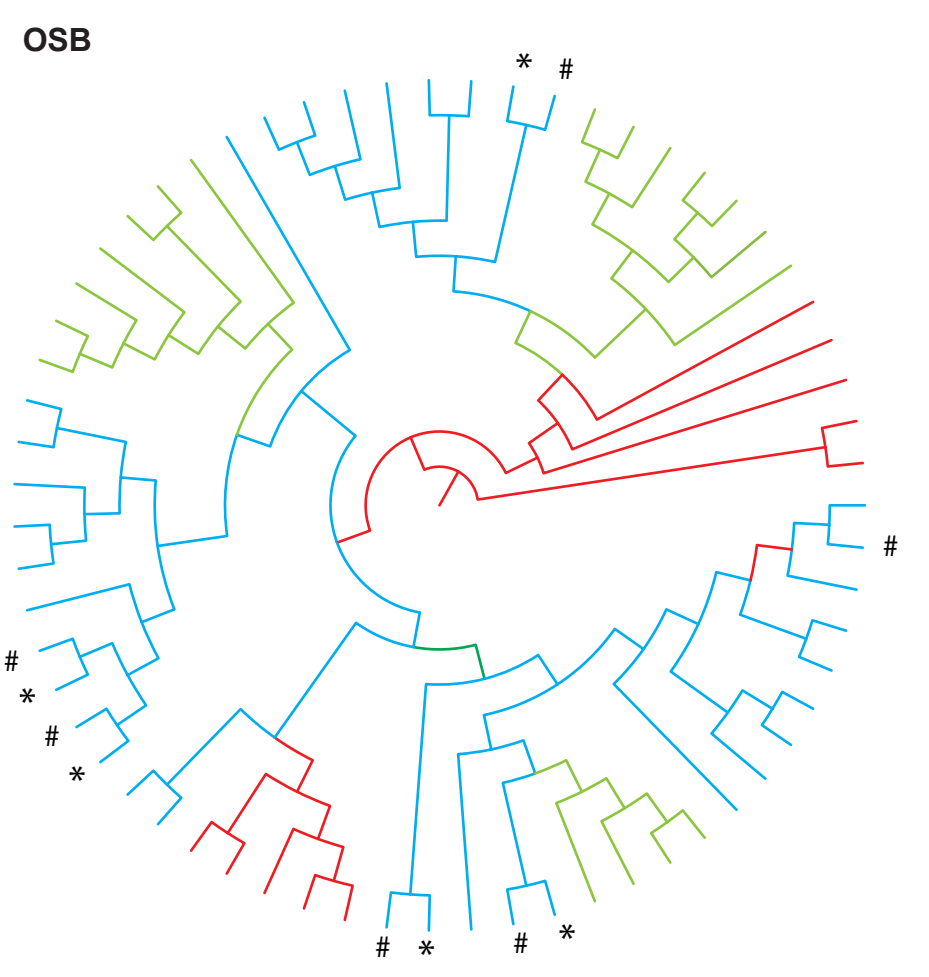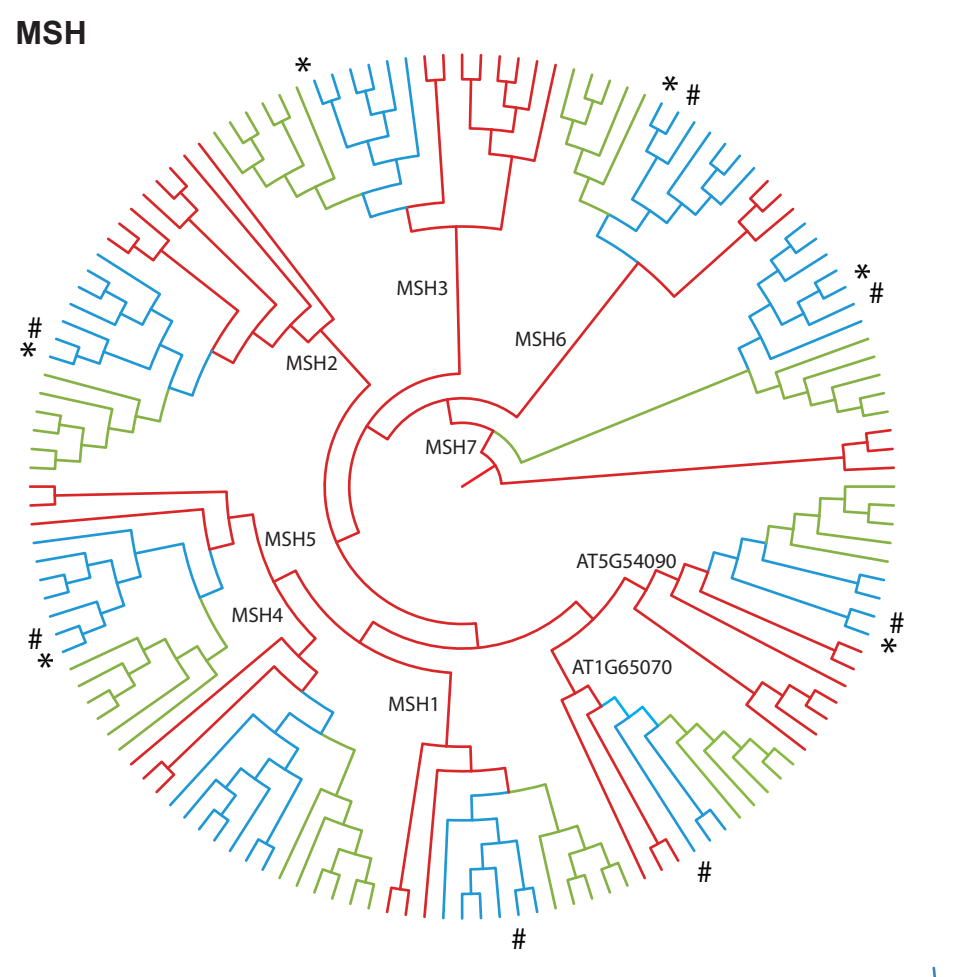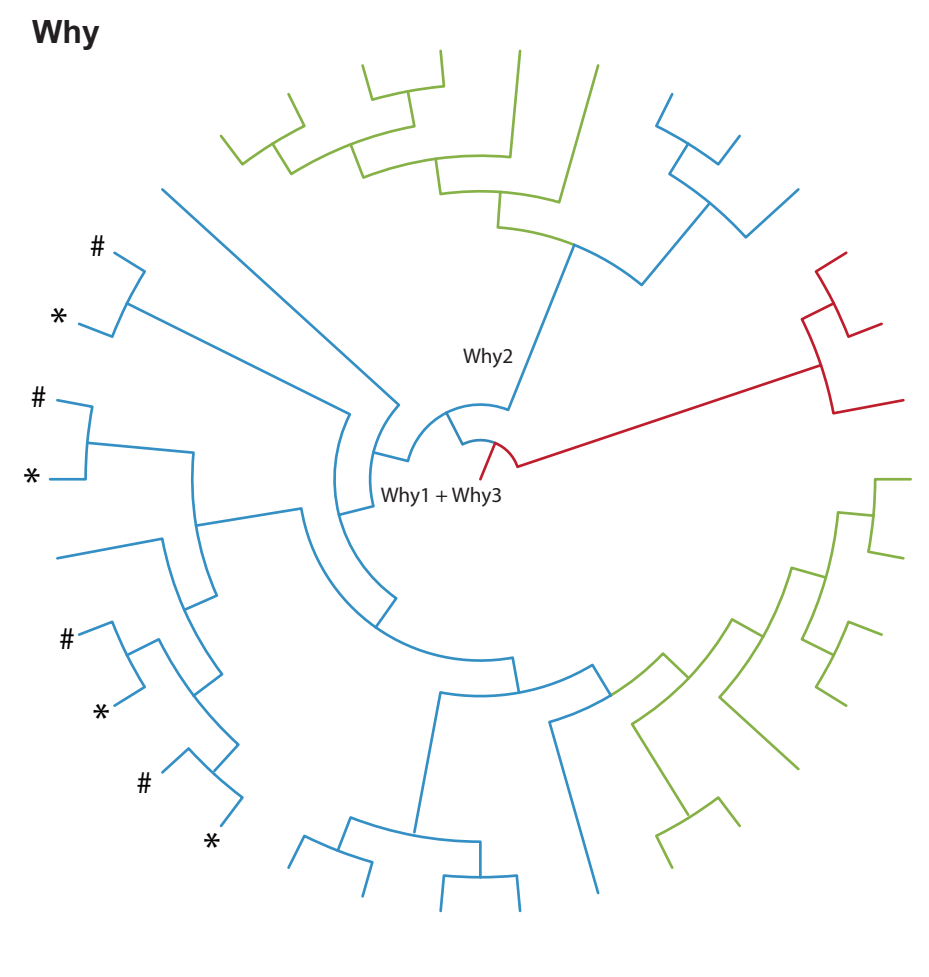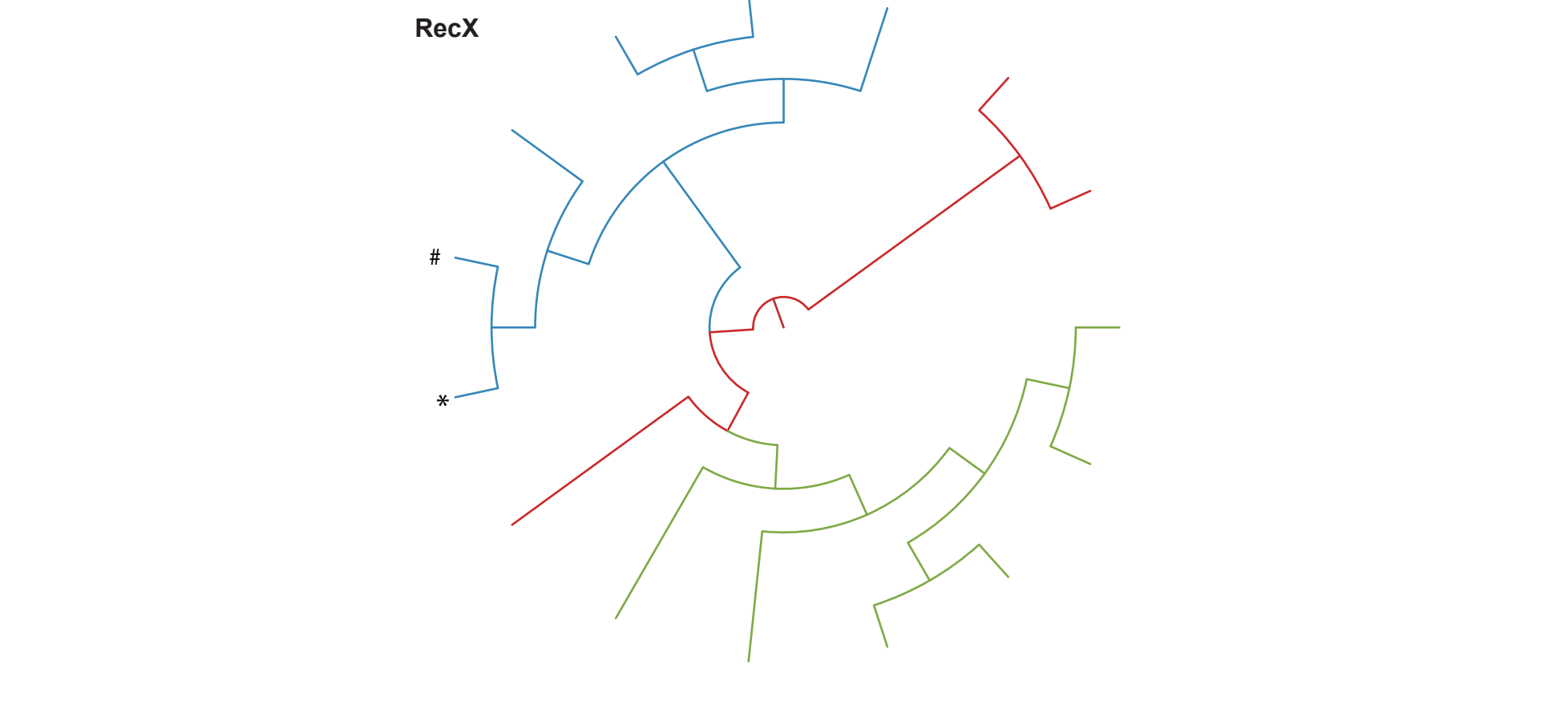

Supplement: S1 Fig — * and # indicate the position of cycad species i.e., Cycas debaoensis and Cycas panzhihuaensis, respectively. (PDF) [file pone.0255091.s001.pdf]
