# Supplementary material for: The complete mitochondrial genome of Cycas debaoensis revealed unexpected static evolution in gymnosperm species
Source: PLoS One. 2021 Jul 22;16(7):e0255091. doi: 10.1371/journal.pone.0255091 (PMC8297867; doi:10.1371/journal.pone.0255091)
Supplement: Attachment — Submitted filename: Responce to reviewer.docx [file pone.0255091.s009.docx]

Reviewer #1: Review of “The complete mitochondrial genome of *Cycas debaoensis* revealed unexpected static evolution in gymnosperm species”

It is nice to have the second *Cycas* species sequenced for the mitochondrial genome, as it provides data to gain insights into mitogenome evolution in closely related species in this early diverging lineage of gymnosperms. The authors have done a good job of describing the genome structure and content, and have also done a somewhat informative comparison on genome content between cycads and other land plants.

One major point that the authors have failed to appreciate is that the second Cycas mitogenome provides an excellent opportunity to demonstrate the amplitude of genome rearrangement among closely related species in different lineages of land plants, where the genome embarked on a path of radical structural evolution, among all eukaryotes. Until now, more than one species for some genera have been sequenced in liverworts (*Calypogeia*), mosses (*Bartramia, Climacium, Niphotrichum, Orthotrichum, Racomitrium*, and *Stoneobryum*), hornworts (*Anthoceros*), and monocot angiosperms (*Oryza*) and eudicot angiosperms (*Beta, Brassica, Corchorus, Glycine, Gossypium, Helianthus, Nicotiana, Populus, Salix, Senna, Solanum, Sorbus, Spondias*, and *Trifolium*). The authors could have done an inter-species mitogenome rearrangement, intron, repeat sequence comparison among all these genera, and then plotted the result along a phylogenetic gradient of land plants. Any emerging pattern will be more meaningful than the sort of comparison they have reported on genome rearrangement, gene and intron content, and repeat in the manuscript.

Response: We have done an inter-species mitogenome rearrangement, intron, repeat sequence comparison as suggested by the reviewer. Inter-species mitogenome rearrangement analysis includes 46 taxa with enough representation from all the major lineages of land plants. Number of repeats from each observed taxa are also mentioned along a phylogenetic gradient of land plants (Fig3.eps). Furthermore, distribution pattern of 26 ancestral seed plant introns among major land plant groups has also been investigated and discussed (Fig2.eps).

A minor point that the authors could have done more analysis is about RNA editing (Lines 413-415 in their manuscript). They should check to see how their results conform to the editing pattern shaped by selection as suggested by Jobson and Qiu (2008).

Response: we explored whether the editing frequencies in gymnosperm species are shaped by selection constraint on genes as suggested by Jobson and Qiu (2008). Three gymnosperm taxa included in the analysis were *C. debaoensis*, *G. biloba* and *T. cuspidata*. We found that membrane-bounded and soluble protein coding genes have experienced similar selective pressures as there is no clear pattern among editing efficiency and gene substitution rates (*d_N_* and *d_S_* vs. editing efficiency %) of three observed gymnosperm taxa (S7 Table).

All the relevant details are mentioned in revised manuscript.
